# *Agrimonia pilosa* Ledeb. Ameliorates Hyperglycemia and Hepatic Steatosis in Ovariectomized Rats Fed a High-Fat Diet

**DOI:** 10.3390/nu12061631

**Published:** 2020-06-01

**Authors:** Hwan-Hee Jang, Ji Hyun Bae, Mi-Ju Kim, Mi Young Park, Haeng Ran Kim, Young-Min Lee

**Affiliations:** 1 Functional Food Division, National Institute of Agricultural Sciences, Rural Development Administration, Wanju 55365, Korea; rapture19@korea.kr (H.-H.J.); godheaven@hanmail.net (J.H.B.); miju0522@gmail.com (M.-J.K.); pmy1222@gmail.com (M.Y.P.); kimhrr@korea.kr (H.R.K.); 2 Division of Applied Food System, Major of Food and Nutrition, Seoul Women’s University, Seoul 01797, Korea

**Keywords:** *Agrimonia pilosa*, hyperglycemia, hepatic steatosis, high-fat diet, ovariectomy.

## Abstract

Estrogen deficiency is associated with obesity, dyslipidemia, and increased insulin resistance in postmenopausal women. An efficient therapeutic agent prevents or improves postmenopausal conditions induced by estrogen deficiency. Here, we investigated the effects of aqueous *Agrimonia pilosa* Ledeb. extract on glucose and lipid metabolism in ovariectomized rats fed a high-fat diet (HFD). Female Sprague-Dawley rats were sham-operated or ovariectomized, and 3 weeks later were assigned to the following groups: sham-operated + HFD (S); ovariectomized + HFD (OVX); and ovariectomized + HFD with 0.5% *A. pilosa* aqueous extract (OVX + 0.5A) groups. Ovariectomy significantly increased body weight and dietary intake relative to the S group. However, *A. pilosa* treatment did not significantly affect weight gain or dietary intake. Blood triacylglycerol, total cholesterol, and low-density lipoprotein cholesterol levels tended to decrease in the *A. pilosa*-supplemented group. Blood glucose levels were significantly lower in the OVX + 0.5A group than those in the OVX group. Blood adiponectin and insulin concentrations increased significantly after *A. pilosa* treatment in the ovariectomized group. *A. pilosa* supplementation tended to decrease liver weights and prevented lipid accumulation. These effects correlated with reduced hepatic expression of lipogenesis-related genes (fatty acid synthase, acetyl-coenzyme A carboxylase alpha, and 3-hydroxy-3-methylglutaryl-coenzyme A reductase). Therefore, *A. pilosa* may improve metabolic disorders in ovariectomized rats.

## 1. Introduction

Postmenopausal women often show marked increases in the incidence of many chronic diseases such as metabolic syndrome, cardiovascular disease, cognitive impairment, and osteoporosis [1,2,3]. Estrogen deficiency is also related to a higher risk of progressing to liver steatosis in postmenopausal women [4]. Hormone-replacement therapy (HRT) can be administered for chronic-disease prevention and menopausal-symptom improvement in postmenopausal women [5,6]. However, data from many studies showed that long-term HRT is not a favorable solution for treating postmenopausal women because it poses increased risks for stroke and breast cancer [7,8]. Consequently, complex risk-versus-benefit patterns in HRT are making postmenopausal women seek alternative treatments for their symptoms (particularly phytoestrogens).

Phytoestrogens are naturally occurring plant-derived compounds found in diverse common foods such as soybeans, fruits, and vegetables [9,10] in the form of polyphenols, flavonoids, and isoflavonoids. Because they have a structure that is similar to mammalian estrogen, they can bind to human estrogen receptors. Phytoestrogens are known to exert various beneficial actions through the modulation of transcriptional activity of nuclear receptors and nuclear-receptor-independent mechanisms. Natural estrogens were reported to improve estrogen-deficiency-related metabolic alterations in glucose and lipid metabolism [11,12,13]. Thus, it is important to identify unknown food components with estrogenic activity and develop products for postmenopausal women as alternatives to HRT.

*Agrimonia pilosa* Ledeb. is a medicinal plant characterized by anticancer, antioxidant, and anti-inflammatory activities [14,15,16]. Furthermore, an in vitro study suggested that *A. pilosa* extracts had estrogen-like activity primarily mediated through estrogen receptors in MCF-7 cells [17]. Our previous studies showed that *A. pilosa* extracts improved insulin resistance in C2C12 myotubes treated with fatty acid, and in rats fed a high-fat diet (HFD) [18,19]. However, the ameliorating effects of *A. pilosa* supplementation in an ovariectomized model have not been investigated. Therefore, we hypothesized that aqueous *A. pilosa* extract, suggested to have phytoestrogen activities, could improve estrogen-deficiency-related metabolic dysregulation in ovariectomized rats. The objective of this study was to investigate the beneficial effects of aqueous *A. pilosa* extract on hyperglycemia and hepatic steatosis induced by estrogen deficiency in an experiment model of postmenopausal metabolic syndrome.

## 2. Materials and Methods

### 2.1. Preparation of Aqueous Extract from A. pilosa

Aerial parts of *A. pilosa*, produced in Yeongcheon, Korea, were purchased from the Kyungdong market (Seoul, Korea) in dried form, and identified by the Classification and Identification Committee of the Korea Institute of Oriental Medicine (KIOM). A voucher specimen (KIOM109-122Aa) was deposited in the herbarium of the Department of Herbal Resources Research in KIOM. Dried plants were extracted with hot water as described previously [17], and water filtrates were freeze-dried. The lyophilized extracts were stored at −20 °C until use.

### 2.2. Experiment Animals and Diets

All animal protocols were approved by the Institutional Animal Care and Use Committee of the National Academy of Agricultural Science (reference number: NAS-1007). Female Sprague-Dawley rats (7 weeks old) were obtained from Orient Bio Inc. (Seongnam, Korea), housed at a constant temperature of 22 °C, and exposed to a 12/12 h (light/dark) cycle. After adaptation for 1 week, the rats were subjected to a sham operation or ovariectomized. After 3 weeks, the rats were assigned to the three following groups: sham-operated + HFD (S group); ovariectomized + HFD (OVX group); and ovariectomized + HFD with 0.5% aqueous *A. pilosa* extract (OVX + 0.5A group). Aqueous *A. pilosa* extract was mixed with the diet. The rats in each group were maintained on these experimental diets for 4 weeks. The composition of each diet is shown in Supplementary Table S1. Water and food were provided ad libitum, and food intake and body weights were measured. Food-efficiency ratio (FER) was calculated using the following formula: weight gain (g/day)/food intake (g/day).

### 2.3. Blood and Tissue Collection for Biochemical Analysis

After the rats were on the experimental diets for 4 weeks, blood samples were obtained via heart puncture under anesthesia using carbon dioxide (CO_2_) gas, and serum was collected by centrifugation at 1500× *g* for 15 min at 4 °C and stored at −70 °C until analysis. Liver and adipose fat tissue was collected as described previously [19].

### 2.4. Biochemical Serum Analysis 

Serum glucose, triacylglycerol (TG), total cholesterol, and high-density lipoprotein cholesterol (HDL cholesterol) were determined using enzyme assay kits obtained from Asan Pharmaceutical (Yongin, Korea), according to the manufacturer’s protocols. Low-density lipoprotein (LDL) cholesterol level was calculated by the Friedewald equation [20] that estimates LDL cholesterol as (total cholesterol) – (HDL cholesterol) – (TG/5) in mg/dL. Free fatty acids were measured using a commercial kit (Zen-Bio Inc., Durham, NC, USA). The serum concentrations of adiponectin, leptin, and insulin were determined using a Rat Endocrine Lincoplex Kit (catalog number RENDO-85K, Linco Research, St. Charles, MO, USA) for Luminex according to the manufacturer’s protocol. The assays were performed in duplicate (n = 9 or 10).

### 2.5. Real-Time Quantitative Reverse Transcription PCR

Total RNA was isolated from hepatic tissue using an RNeasy Microarray Tissue Kit (Qiagen, Valencia, CA, USA), and RNA integrity (RIN > 9.0) was assessed using a Bioanalyzer 2100 (Agilent Technologies, Santa Clara, CA, USA) as described previously [19]. The Rat Fatty Liver PCR Array (SA Biosciences, Frederick, MD, USA) was used to profile differentially expressed genes in accordance with the manufacturer’s instructions. The complete list of genes assayed on the array is provided on the manufacturer’s website (https://geneglobe.qiagen.com/product-groups/rt2-profiler-pcr-arrays). Data were normalized using lactate dehydrogenase A (LDH) as endogenous control, and fold changes in expression were calculated using the 2^−ΔΔCT^ method.

### 2.6. Liver Histology

Liver tissue was fixed with 10% neutral buffered formalin and embedded in paraffin. Sections were cut and stained with hematoxylin and eosin (H&E). Images were obtained using an Olympus AX 70 camera (Center Valley, PA, USA). Hepatic steatosis was graded as 0 (fatty hepatocytes occupying <5%), 1 (fatty hepatocytes occupying 5–33%), 2 (fatty hepatocytes occupying 34–66%), or 3 (fatty hepatocytes occupying >66%), in terms of the percentages of hepatic lipids [21].

### 2.7. Statistical Analysis

Data are shown as the mean ± standard error (SE). Statistical comparisons were performed by one-way analysis of variance followed by Duncan’s multiple-range tests. Differences were considered statistically significant when the *p*-value was less than 0.05. All statistical analyses were conducted using SPSS software, version 21.0 (IBM Corp., Armonk, NY, USA).

## 3. Results

### 3.1. Effects of A. pilosa on Body Weight, Food Intake, Food-Efficiency Ratio (FER), and Organ Weight

Body weight, food intake, and FER are presented in Table 1. After 4 weeks on the assigned diets, the rats in the ovariectomized groups weighed significantly more than those in the S group. However, there was no significant difference between the OVX and OVX + 0.5A groups. Although dietary intake was significantly increased in the ovariectomized rats, food intake did not significantly differ between the OVX and OVX + 0.5A groups throughout the experiment period. *A. pilosa* supplementation did not significantly change body weight and food intake.

Supplementary Table S2 shows the liver- and adipose-fat-tissue weights of the rats. The OVX group showed significant increases in liver and adipose-fat weights compared to those of the S group. Although there was no statistical significance, the OVX + 0.5A group showed a tendency toward decreased liver weight compared to that found for the OVX group.

### 3.2. Effects of A. pilosa on Serum Lipid Levels

The levels of serum TG, cholesterol, free fatty acids (FFAs), and atherogenic index (AI) are shown in Table 2. Serum total cholesterol and LDL cholesterol levels showed a tendency to decrease in the *A. pilosa*-supplemented group. In the OVX + 0.5A group, serum FFA concentration significantly decreased compared to that of the S group. In addition, the AI value was significantly lower in the OVX + 0.5A group than that in the OVX group.

### 3.3. Effects of A. pilosa on Serum Metabolic Parameters

Serum metabolic parameters such as insulin, glucose, leptin, and adiponectin did not show significant differences between the S and OVX groups. *A. pilosa* supplementation significantly affected fasting blood glucose levels (Table 3). Serum glucose concentration decreased by 26% in the OVX + 0.5A group compared that in the OVX group. Serum insulin and adiponectin levels were higher in the OVX + 0.5A than those in the OVX group. However, *A. pilosa* supplementation did not significantly affect serum leptin concentration.

### 3.4. Effect of A. pilosa on Hepatic Steatosis

**Figure 1:**
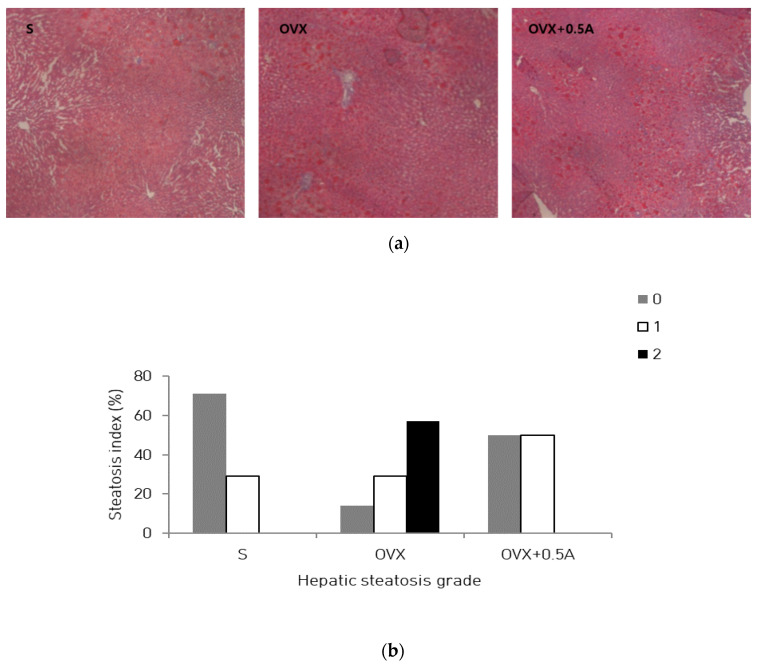
Effect of *A. pilosa* supplementation on hepatic steatosis in OVX rats fed an HFD. (**a**) Photomicrographs of hematoxylin and eosin-stained liver from representative rats in (**left**) S, (**middle**) OVX, and (**right**) OVX + 0.5A groups (×400). (**b**) Percentages of rats with each hepatic-steatosis grade (0–3 scale) for each group. Hepatic steatosis graded as 0 (fatty hepatocytes occupying <5% of hepatic lipids), 1 (fatty hepatocytes occupying 5–33%), 2 (fatty hepatocytes occupying 34–66%), or 3 (fatty hepatocytes occupying >66%). Abbreviations: S, sham-operated + HFD; OVX, ovariectomized + HFD; OVX+0.5A, ovariectomized + HFD with 0.5% aqueous *A. pilosa* extract.

The effect of *A. pilosa* supplementation on lipid accumulation is shown in Figure 1. H&E staining demonstrated that few lipid droplets in the liver were observed when the rats were administered an HFD for 4 weeks without ovariectomy (Figure 1a, left), whereas the ovariectomized rats fed an HFD showed noticeable lipid-droplet formation in the liver (Figure 1a, middle). A*. pilosa* supplementation decreased lipid-droplet formation when compared with ovariectomized rats fed an HFD (Figure 1a, right).

### 3.5. Effects of A. pilosa Supplementation on Lipid-Metabolism-Related Gene Expression

**Figure 2:**
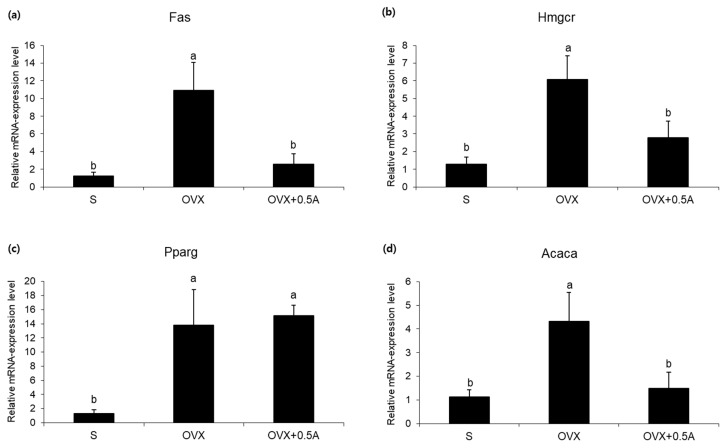
Effects of *A. pilosa* on differential expression of lipogenesis-related genes (**a**–**d**) in fatty liver PCR array. Fas, fatty acid synthase; Hmgcr, 3-hydroxy-3-methylglutaryl-coenzyme A reductase; Pparg, peroxisome proliferator-activated receptor γ; Acaca, acetyl-coenzyme A carboxylase alpha; S, sham-operated + HFD; OVX, ovariectomized + HFD; OVX+0.5A, ovariectomized + HFD with 0.5% aqueous *A. pilosa* extract. Data shown were normalized to C_t_ value of lactate dehydrogenase A (LDH), and are expressed as mean ± SE of 4 rats per group. Values with different superscripted letters (a,b) within same graph were significantly different at *p* < 0.05, as determined by Duncan’s multiple-range test.

To clarify the mechanisms through which *A. pilosa* supplementation resulted in an improvement of fatty liver, the relative mRNA levels of fatty liver-related genes were investigated. As shown in Figure 2, the expression levels of lipogenesis-metabolism-related genes, including acetyl-coenzyme A carboxylase alpha (Acaca), fatty acid synthase (Fas), and 3-hydroxy-3-methylglutaryl-coenzyme A reductase (Hmgcr) were significantly lower in the OVX + 0.5A group than those in the OVX group. However, *A. pilosa* supplementation did not suppress expression of peroxisome proliferator-activated receptor γ (Pparg).

## 4. Discussion

The present study confirmed that *A. pilosa* has antihyperglycemic and hepatoprotective effects against abnormal metabolism induced by estrogen deficiency in rats fed an HFD. In this study, the OVX group had significantly higher body and liver weights, and elevated fasting blood glucose levels compared to those reported for the S group. However, dietary *A. pilosa* supplementation significantly decreased blood glucose levels, while elevating blood adiponectin and insulin concentrations. Serum TC and LDL-C levels in the OVX + 0.5A group were reduced, although differences were not statistically significant. Consequently, AI was significantly lower in the OVX + 0.5A group than that in the OVX group. The effects on serum lipids observed in the OVX + 0.5A group were associated with reduced hepatic steatosis relative to that seen in the OVX group. Hepatic fat accumulation and gene expression related to the regulation of lipid synthesis were suppressed by *A. pilosa* supplementation.

OVX rats fed an HFD showed excessive hepatic TG accumulation. Liver-fat accumulation was markedly greater in HFD-fed estrogen-receptor-deficiency model rats than that in HFD-fed control rats [22]. However, *A. pilosa* supplementation tended to reduce liver weight and prevent hepatic lipid accumulation in ovariectomized rats fed an HFD. Hepatic lipid turnover is regulated through enzymes and transcription factors related to lipogenesis and fat oxidation [23]. To clarify the mechanisms through which *A. pilosa* resulted in improved fatty liver, the relative mRNA expression levels of fatty liver-related genes were investigated. *A. pilosa* significantly decreased the mRNA expression levels of lipid-anabolism-related genes including Acaca, Fas, and Hmgcr, whereas it showed no effect on genes involved in ß-oxidation such as acyl-CoA-dehydrogenase and carnitine palmitoyltransferase 1A (Supplementary Table S3). Acaca, Fas, and Hmgcr catalyze rate-limiting steps in fatty acid and cholesterol synthesis. Therefore, suppressing the expression of these genes could have helped prevent hepatic steatosis in the ovariectomized rats. Previous findings suggested that increased hepatic steatosis is strongly related to metabolic abnormalities, including insulin resistance, suggesting that the inhibition of hepatic fat accumulation by *A. pilosa* could help to prevent hyperglycemia [24].

In this study, the OVX group showed a tendency to increase fasting blood glucose levels compared to the S-group rats, implying that glucose metabolism in postmenopausal women may be impaired by estrogen deficiency. We observed that the blood glucose levels of rats in the OVX + 0.5A group were significantly lower than those in the OVX group. In addition, *A. pilosa* effectively decreased the concentration of serum FFAs, indicating that *A. pilosa* could ameliorate glucose metabolism in ovariectomized rats. Elevated concentrations of blood FFAs, also known as nonesterified fatty acids, are related to insulin resistance [25]. Because insulin suppresses FFA release from adipose tissue, insulin-resistant adipose tissue is associated with increased blood FFA levels. Therefore, our results suggested that *A. pilosa* may be used to improve hyperglycemia after menopause.

The developmental mechanism of nonalcoholic fatty liver disease (NAFLD) is considered to be primarily related to insulin resistance via the roles of inflammatory cytokines and adipocytokines (e.g., adiponectin and leptin) [24]. Specifically, blood adiponectin levels have been negatively correlated with histological parameters and metabolic indicators in patients with NAFLD [26]. Adiponectin, which is an antidiabetic adipocytokine, has been reported to be significantly lower in NAFLD patients [27,28]. It is well known that adiponectin performs insulin-sensitizing actions such as inhibiting gluconeogenesis, blocking de novo lipogenesis, and activating fatty acid oxidation in the liver [29]. A negative association has also been found between serum adiponectin concentration and the degree of hepatic steatosis. Adiponectin acts via two distinct adiponectin receptors (ADNRs), i.e., ADNR-1 and ADNR-2, and insulin sensitizers, including rosiglitazone, exert activity by upregulating hepatic ADNRs [30]. In the present study, although *A. pilosa* did not affect the expression of ADNRs in the liver (Supplementary Table S3), *A. pilosa* supplementation markedly ameliorated hepatic steatosis by increasing blood adiponectin levels.

OVX-induced metabolic disturbance is associated with impaired glucose homeostasis. Moreover, ovariectomy and diabetes exaggerated insulin resistance while reducing adiponectin levels in serum and adipose tissue [31]. Generally, adiponectin improves insulin sensitivity, which is associated with low blood insulin and the homeostasis model assessment of insulin resistance (HOMA-IR) index. However, our results showed that both adiponectin and insulin concentrations in the OVX + 0.5A group were significantly elevated compared to those in the OVX group. Rao et al. showed that adiponectin can increase insulin secretion in MIN6 cells [32]. Moreover, globular adiponectin supplementation increased insulin secretion and decreased glucose levels in rats with Type 2 diabetes and NAFLD [33]. Several medicinal-herb extracts showed hypoglycemic effects that promoted insulin secretion, in accordance with our results [34,35]. *A. pilosa* extracts improved insulin resistance in rats fed an HFD, while decreasing blood insulin and HOMA-IR, in our previous study [19]. Further studies are needed to understand the molecular mechanisms of the insulin secretory effect of *A. pilosa* aqueous extract.

In our previous study, we reported estrogen-like activity in aqueous *A. pilosa* extract containing apigenin-glucuronide and luteolin-glucuronide as major flavonoids [17]. These conjugated flavonoids were suggested to be the most active constituents in herbal extracts with estrogenic activity [36]. A recent study reported that long-term supplementation of low doses of apigenin could improve HFD-induced comorbidity through metabolic and transcriptional regulation in the liver [37]. It is predicted that the ameliorating effects of *A. pilosa* supplementation on hyperglycemia and hepatic steatosis are likely attributable to these flavonoids contained within the plant. However, a limitation of the present study was that we did not analyze the estrogenic activities of *A. pilosa* such as estrogen-receptor activation and the uterotrophic effect in the experiment model of postmenopausal metabolic syndrome. Further studies to isolate and characterize the functionality of each compound derived from *A. pilosa* are needed. It is also necessary to investigate the impact of *A. pilosa* on postmenopausal women’s health.

## 5. Conclusions

In conclusion, *A. pilosa* augmented serum insulin and adiponectin levels, prevented hyperglycemia, decreased serum FFAs, regulated hepatic expression of lipogenesis-related genes, and improved hepatic steatosis in ovariectomized rats. These results suggested that *A. pilosa* may be a potential healthful food option for postmenopausal women who are characterized by estrogen-deficiency-associated metabolic abnormalities.

## Figures and Tables

**Table 1:** Effects of *A. pilosa* on body weight, weight gain, food intake, and FER of rats.

	S ^1^	OVX	OVX + 0.5A
Initial body weight (BW), g	276.2 ± 2.8 ^2,NS,3^	276.2 ± 2.8	276.2 ± 2.8
OVX (at 0 week) BW, g	288.5 ± 6.6 ^b^	334.3 ± 6.4 ^a^	338.0 ± 8.4 ^a^
Final BW (after 4 weeks), g	336.6 ± 11.8 ^b^	393.8 ± 9.1 ^a^	389.4 ± 6.1 ^a^
Weight gain (after 4 weeks), g	48.1 ± 8.3 ^NS^	59.5 ± 5.7	58.5 ± 4.6
Intake (for 4 weeks), g/d	18.3 ± 0.7 ^b^	21.0 ± 0.6 ^a^	20.9 ± 0.7 ^a^
FER ^4^ (for 4 weeks)	0.09 ± 0.01 ^NS^	0.10 ± 0.01	0.11 ± 0.01

^1^Abbreviations: S, sham-operated + high-fat diet (HFD); OVX, ovariectomized + HFD; OVX + 0.5A, ovariectomized + HFD with 0.5% aqueous *Agrimonia pilosa* extract; NS, not significant; FER, food-efficiency ratio. ^2^Data shown expressed as mean ± standard error (SE) of 9–10 rats per group. ^3^Values with different superscripted letters within same row were significantly different at *p* < 0.05, as determined by Duncan’s multiple-range test. NS: *p* ≥ 0.05. ^4^FER = weight gain (g/day)/food intake (g/day).

**Table 2:** Effect of *A. pilosa* on serum lipid levels.

	S ^1^	OVX	OVX + 0.5A
TG, mg/dL	71.80 ± 2.14 ^2,NS,3^	69.69 ± 12.18	60.00 ± 2.06
Total cholesterol, mg/dL	115.23 ± 6.22 ^NS^	163.85 ± 36.95	121.17 ± 12.49
HDL cholesterol, mg/dL	18.08 ± 1.11 ^b^	32.24 ± 3.53 ^a^	37.03 ± 3.28 ^a^
LDL cholesterol, mg/dL	81.25 ± 7.78 ^NS^	120.84 ± 35.09	69.21 ± 12.74
Free fatty acid, μM	205.26 ± 10.82 ^a^	192.09 ± 35.53 ^a,b^	128.97 ± 18.63 ^b^
AI ^4^	5.55 ± 1.14 ^a^	5.97 ± 1.45 ^a^	1.89 ± 0.35 ^b^

^1^Abbreviations: S, sham-operated + HFD; OVX, ovariectomized + HFD; OVX+0.5A, ovariectomized + HFD with 0.5% aqueous *A. pilosa* extract; TG, triacylglycerol; HDL, high-density lipoprotein; LDL, low-density lipoprotein; NS, not significant; AI, atherogenic index. ^2^Data shown expressed as mean ± SE of 9–10 rats per group. ^3^Values with different superscripted letters within same row were significantly different at *p* < 0.05, as determined by Duncan’s multiple-range test. NS: *p* ≥ 0.05. ^4^ AI = [(Total cholesterol)–(HDL cholesterol)]/(HDL cholesterol).

**Table 3:** Effect of *A. pilosa* on serum metabolic parameters.

	S ^1^	OVX	OVX + 0.5A
Insulin, pM	322.05 ± 35.51 ^2,b,3^	462.88 ± 58.43 ^b^	765.33 ± 104.24 ^a^
Glucose, mg/dL	163.12 ± 12.58 ^a,b^	193.86 ± 18.00 ^a^	143.70 ± 8.53 ^b^
Leptin, pM	548.9 ± 59.9 ^2,NS,3^	728.3 ± 158.3	736.7 ± 54.9
Adiponectin, µg/mL	13.34 ± 1.44 ^b^	11.99 ± 1.57 ^b^	20.75 ± 2.73 ^a^

^1^Abbreviations: S, sham-operated + HFD; OVX, ovariectomized + HFD; OVX + 0.5A, ovariectomized + HFD with 0.5% aqueous *A. pilosa* extract; NS, not significant. ^2^Data shown expressed as mean ± SE of 9–10 rats per group. ^3^Values with different superscripted letters within same row were significantly different at *p* < 0.05, as determined by Duncan’s multiple-range test. NS: *p* ≥ 0.05.

## Supplementary Materials

The following are available online at https://www.mdpi.com/2072-6643/12/6/1631/s1. Table S1: compositions of experimental diets; Table S2: liver- and fat-tissue weights of rats; Table S3: expression of fatty acid β-oxidation-related genes and adiponectin receptors.
