# Supplementary material for: Agrimonia pilosa Ledeb. Ameliorates Hyperglycemia and Hepatic Steatosis in Ovariectomized Rats Fed a High-Fat Diet
Source: Nutrients. 2020 Jun 1;12(6):1631. doi: 10.3390/nu12061631 (PMC7352636; doi:10.3390/nu12061631)
Supplement: Supplementary file 1 [file nutrients-12-01631-s001.zip › Supplementary_Table_1.docx]

**Table S1.** Compositions of the experimental diets (g/kg diet)

|  | S^1^ and OVX | OVX+0.5A |
| --- | --- | --- |
| Cornstarch | 470.692 | 465.692 |
| Casein | 140.000 | 140.000 |
| Sucrose | 100.000 | 100.000 |
| Beef tallow | 180.000 | 180.000 |
| Cholesterol | 10.000 | 10.000 |
| Fiber | 50.000 | 50.000 |
| Mineral mixture^2^ | 35.000 | 35.000 |
| Vitamin mixture^3^ | 10.000 | 10.000 |
| L-cysteine | 1.800 | 1.800 |
| Choline bitartrate | 2.500 | 2.500 |
| TBHQ^4^ | 0.008 | 0.008 |
| Aqueous *A. pilosa* extract | - | 5.000 |

^1^ Abbreviations: S, sham-operated + HFD; OVX, ovariectomized + HFD; OVX+0.5A, ovariectomized + HFD with 0.5% aqueous *A. pilosa* extract

^2^ Mineral mixture: AIN-93M mineral mixture (ICN, CA, USA)

^3^ Vitamin mixture: AIN-93VX vitamin mixture (ICN, CA, USA)

^4^ TBHQ: tert-butylhydroquinone
