# Supplementary material for: Agrimonia pilosa Ledeb. Ameliorates Hyperglycemia and Hepatic Steatosis in Ovariectomized Rats Fed a High-Fat Diet
Source: Nutrients. 2020 Jun 1;12(6):1631. doi: 10.3390/nu12061631 (PMC7352636; doi:10.3390/nu12061631)
Supplement: Supplementary file 1 [file nutrients-12-01631-s001.zip › Supplementary_Table_2.docx]

**Table S2.** Liver and fat tissue weights of rats

|  | S**^1^** | OVX | OVX+0.5A |
| --- | --- | --- | --- |
| Liver, g | 10.84±0.42^2,b,3^ | 12.69 ± 0.41^a^ | 12.21 ± 0.59^ab^ |
| Adipose fat, g | 20.55±2.20^b^ | 25.51 ± 2.14^a^ | 26.85 ± 1.47^a^ |

^1^ Abbreviations: S, sham-operated + HFD; OVX, ovariectomized + HFD; OVX+0.5A, ovariectomized + HFD with 0.5% aqueous *A. pilosa* extract

^2^ The data shown are expressed as the mean ± S.E. of 9–10 rats per group.

^3^ Values with different superscripted letters within the same row are significantly different at p < 0.05, as determined by Duncan's multiple-range test. NS: p ≥ 0.05
