# Supplementary material for: Agrimonia pilosa Ledeb. Ameliorates Hyperglycemia and Hepatic Steatosis in Ovariectomized Rats Fed a High-Fat Diet
Source: Nutrients. 2020 Jun 1;12(6):1631. doi: 10.3390/nu12061631 (PMC7352636; doi:10.3390/nu12061631)
Supplement: Supplementary file 1 [file nutrients-12-01631-s001.zip › Supplementary_Table_3.docx]

**Table S3.** Expression of fatty acid ß-oxidation related genes and adiponectin receptors in a fatty liver PCR array

|  | S**^1^** | OVX | OVX+0.5A |
| --- | --- | --- | --- |
| Acyl-Coenzyme A dehydrogenase, long-chain | 1.20 ± 0.36^2,NS,3^ | 2.58 ± 0.34 | 2.37 ± 0.97 |
| Carnitine palmitoyltransferase 1a | 1.11 ± 0.27 ^NS^ | 1.67 ± 0.39 | 1.84 ± 0.94 |
| Adiponectin receptor 1 | 1.33 ± 0.63 ^NS^ | 3.35 ± 1.27 | 2.33 ± 0.89 |
| Adiponectin receptor 2 | 1.11 ± 0.29 ^NS^ | 2.15 ± 0.61 | 1.09 ± 0.53 |

^1^ Abbreviations: S, sham-operated + HFD; OVX, ovariectomized + HFD; OVX+0.5A, ovariectomized + HFD with 0.5% aqueous *A. pilosa* extract, NS, not significant

^2^ The data shown are normalized to C_t_ value of LDH and are expressed as the mean ± S.E. of 4 rats per group.

^3^ Determined by Duncan's multiple-range test
